# Genetic Diversity and Population Structure Analysis of *Luhua chickens* Based on Genome-Wide Markers

**DOI:** 10.3390/ani15142071

**Published:** 2025-07-14

**Authors:** Qianwen Yang, Wei Han, Jun Yan, Chenghao Zhou, Guohui Li, Huiyong Zhang, Jianmei Yin, Xubin Lu

**Affiliations:** 1 College of Mathematical Science, Yangzhou University, Yangzhou 225009, China; yzuyqw@163.com (Q.Y.); junyan@yzu.edu.cn (J.Y.); 2 Jiangsu Institute of Poultry Science, Yangzhou 225611, China; hanwei830@163.com (W.H.); reddsbhj3@163.com (C.Z.); sahui2008@163.com (G.L.); zhyong1983@163.com (H.Z.); 15952566122@163.com (J.Y.); 3 College of Animal Science and Technology, Yangzhou University, Yangzhou 225009, China

**Keywords:** *Luhua chicken*, whole-genome resequencing, genetic diversity, population structure

## Abstract

The *Luhua chicken* is an outstanding local breed in China that has been placed under conservation due to the impact of specialized breeding and the widespread adoption of commercial varieties. As such, this study analyzed reproductive traits across three consecutive generations and utilized whole-genome resequencing data from 60 *Luhua chickens* to assess conservation efficacy through genetic diversity, run of homozygosity (ROH) distribution, kinship, and population structure so as to better conserve the breed. The results show that, across generations, the body weight at first egg increased, the age at first egg was delayed, and the egg weight at first laying increased. No significant variations were found in the body weight at 300 d or the total egg number. The key genetic parameters of the polymorphism information content (PIC), expected heterozygosity (HE), observed heterozygosity (HO), and mean identical-by-state (IBS) distance were 0.234, 0.351, 0.277, and 0.782, respectively. The majority of ROHs ranged from 0.5 to 1 Mb, and the inbreeding coefficient based on ROHs was calculated at 0.021. The findings reveal that these traits remained unchanged across the three generations. Our research suggests that optimizing the mating plan of *Luhua chickens* is essential to minimize inbreeding risk. Furthermore, the methodology applied in this study provides a valuable reference for the conservation monitoring of other indigenous chicken breeds.

## Simple Summary

The *Luhua chicken* is the only indigenous Chinese breed with an autosomal feather color trait that enables sex differentiation. As specialized varieties of chicken have been widely bred and used in production, Chinese native breeds have been neglected, leading to issues including small breeding populations, disorganized varieties, and even the risk of extinction. As *Luhua chickens* also face these challenges, they were placed in the National Gene Bank of Local Chicken Breeds in China for preservation in 2003. This article provides a comprehensive assessment of the conservation status of *Luhua chickens* from both phenotypic and molecular perspectives. Six phenotypic traits, including age at first egg, body weight at 300 days, and total egg production, were analyzed across three consecutive generations. The results indicate that the conservation status of *Luhua chickens* remains stable, although a minor inbreeding risk was detected, necessitating strategic conservation adjustments. This study not only serves as a valuable reference for *Luhua chickens*’ conservation but also provides insights into the detection and evaluation of the conservation status of other poultry breeds.

## 1. Introduction

The poultry industry has significantly improved global food security and protein availability. Through extensive domestication and selective breeding, various well-known chicken breeds have been developed, including layer breeds, meat breeds, dual-purpose breeds, and ornamental varieties [1,2,3]. During this process, selectively bred varieties with desirable traits (e.g., feed efficiency, disease resistance, and meat quality) became essential.

In 2020, a total of 114 local chicken breeds, including the Luhua breed, were listed in China’s Livestock and Poultry Genetic Resource Variety List. The Luhua breed is characterized by the distinct ruffled feather trait. The breed originated from Wenshang County, Shandong Province, and has been recognized for its excellent egg-laying performance, desirable carcass traits, and high meat quality, classifying it as both a dual-purpose and ornamental breed [4]. Research on the genetic diversity of this local chicken breed could play an important role in the selection of new chicken breeds in the future [5,6].

In order to safeguard local genetic resources, the *Luhua chicken* was introduced into the National Gene Bank of Local Chicken Breeds (Jiangsu, China); its genealogical records have been meticulously maintained since 2009. Over the past 15 years, 60 genealogies have been established, and conservation efforts have employed a family-based, equal seed selection, random mating method. At the same time each year, artificial insemination and machine incubation were used to produce the next generation of chickens, after which various production performance indicators were recorded. In addition, molecular biology techniques, such as SNP gene chip technology, have been used to periodically monitor the effectiveness of the conservation process, aiming to preserve the original characteristics and features of the local breed [7].

Research on the genetic diversity and population genetic structure of the *Luhua chicken* population is vital to ensure more efficient conservation and utilization of genetic resources in local chicken breeds. In the past, studies on Luhua breed conservation have mainly focused on single-generation phenotypic traits and specific genes, such as feather growth, feather color, and body size [8,9,10], while research on multi-generation whole-genome sequencing for breed conservation has been limited. Previous research has confirmed that inbreeding depression significantly impacts reproductive performance in chickens, influencing traits such as egg weight and egg number, which are closely linked to breeding strategies and genetic conservation [11,12,13]. This study aims to assess the genetic diversity and conservation efficacy of *Luhua chickens* by utilizing whole-genome resequencing and phenotypic trait analysis while also evaluating potential inbreeding risks to inform breeding strategies and genetic conservation efforts [14]. Based on a statistical analysis of phenotypic traits across three consecutive generations, this study examines the genetic diversity and structure of the *Luhua chicken* population in China using SNP data. The findings provide valuable insights and a theoretical foundation for future breeding selection and related efforts.

## 2. Materials and Methods

### 2.1. Ethics Statement

This animal study was approved by the Institutional Animal Care and Use Committee (IACUC) of the Yangzhou University Animal Experiments Ethics Committee under permit number SYXK (Su) 2021-0027. All experimental procedures involving chickens were performed in accordance with the Regulations For The Administration Of Affairs Concerning Experimental Animals approved by the State Council of the People’s Republic of China.

### 2.2. Chicken Population

A total of 3153 females from the 7th to 9th generations of *Luhua chicken* breeding lines were selected from the National Gene Bank of Local Chicken Breeds (Yangzhou, Jiangsu, China) for investigation. The production data for three generations of *Luhua chickens* were recorded, with each generation consisting of 60 families. The generations included 903, 1070, and 1180 individuals, respectively. The body weight at first egg, egg yield, and age at first laying were 1490–1513 g, 195–196, and 162–175 d, respectively. All chickens were healthy and raised under the same housing and diet conditions. In accordance with animal welfare standards, the housing environment was maintained within a thermoneutral temperature range suitable for the birds (20–24 °C), and a light schedule of 16 h of light and 8 h of darkness was provided to support natural behavioral rhythms. Furthermore, the chickens were fed three times daily and had ad libitum access to both feed and water. Phenotypic data of the population were analyzed using R software (v4.3.2) to estimate the means, standard deviations (SDs), and 95% confidence intervals for each trait separately.

### 2.3. Genotypic Data and Quality Control

In this study, 60 healthy *Luhua chickens* were randomly selected for whole-genome sequencing. Blood samples were collected, and DNA was extracted using a TIANamp Blood DNA Kit (Cat. no: 4992208) (Tiangen Biochemical Technology (Beijing) Co., Ltd., Beijing, China). After confirming DNA quality via Polymerase Chain Reaction (PCR), genome resequencing was performed with 10× sequencing depth on the Illumina second-generation sequencing platform using the chicken GRCg7b genome (GCF_016699485.2) as the reference. SNPs were called using GATK software (v4.4.0.0) and filtered based on the following criteria: (1) minor allele frequency (MAF) < 0.05, (2) individual genotype call rate < 99%, and (3) SNP call rate < 90%. After quality control, 1607,248 SNPs remained for further analysis. Then, the SNP density distribution plot on the genome was generated using the CMplot package in R software (v4.3.2), with SNP density statistics calculated in non-overlapping 0.1 Mb windows.

### 2.4. Genetic Diversity Analysis

Population genetic diversity was assessed by calculating MAF, polymorphism information content (PIC), expected heterozygosity (HE), and observed heterozygosity (HO) using Plink software (v1.90).

### 2.5. ROH Statistics and Inbreeding Coefficient Analysis

Genomic runs of homozygosity (ROHs) were detected for each sample, and the distribution, length, and number of ROHs within the population were statistically analyzed. The inbreeding coefficient (FROH) was calculated as the ratio of ROH length to total genome length for each individual. The Fixation index (FST) was calculated using Plink software (v1.90).

### 2.6. Genetic Relationship Analysis

To evaluate kinship between individuals, an identical-by-state (IBS) distance matrix and a genetic relationship matrix (G-matrix) were constructed. The IBS distance matrix was generated using PLINK (v1.90), the G-matrix was built with GCAT software (v1.94.1), and heat maps were created using R software (v4.3.2).

### 2.7. Genetic Structure Analysis

To analyze the genetic structure of *Luhua chickens*, principal component analysis (PCA) was carried out using Plink software (v1.90), and the results were visualized using the ggplot2 package in R software (v4.3.2). Furthermore, to study the family structure thoroughly, the population was classified into different family lines by cluster analysis using genome-wide markers. An implicit genetic distance threshold on the dendrogram was considered to ensure that the identified families were genetically distinguishable and relevant for breeding management purposes.

## 3. Results

### 3.1. Descriptive Statistics

Descriptive statistics for the phenotypic data of *Luhua chickens* are shown in Table 1. No significant differences were found for the body weight at 300 days and the total egg number among the different generations. The mean values of the body weight at first egg, age at first egg, and egg weight were 1487.97 g, 168.40 d, and 38.09 g, respectively.

### 3.2. SNP Distribution Characterization

**Figure 1:**
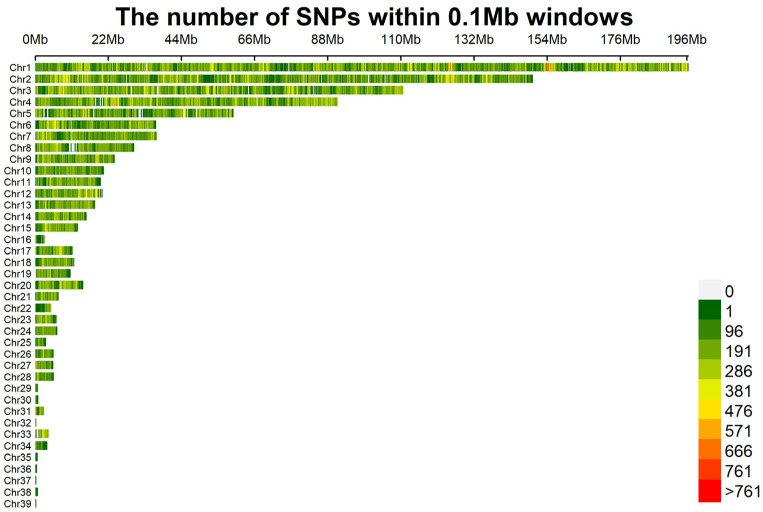
The density distribution of single-nucleotide polymorphisms (SNPs) on chromosomes. After conducting quality control, a total of 1,607,248 SNPs remained. The distribution of the filtered SNPs is displayed over the 39 chromosomes.

A total of 1,628,925 SNPs were detected, 1,607,248 of which were identified as being eligible for the study after quality control. The density distribution of SNPs across chromosomes is shown in Figure 1. Overall, the SNPs were distributed relatively uniformly, with some regions exhibiting notable concentrations.

### 3.3. Genetic Diversity Analysis

**Figure 2:**
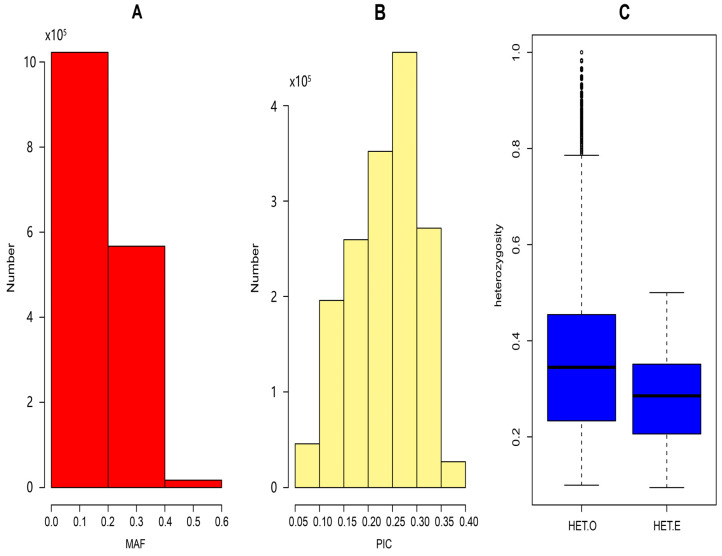
(**A**) Distribution of minor allele frequencies (MAF); (**B**) distribution of polymorphism information content (PIC); (**C**) boxplot of expected heterozygosity (HE) and observed heterozygosity (HO).

The population genetic diversity of *Luhua chickens* is shown in Figure 2. As shown in Figure 2A, MAFs were predominantly concentrated between 0 and 0.2, with a small number exceeding 0.4, indicating that most SNPs have low minor allele frequencies. The PIC values ranged from 0.05 to 0.40, with a mean of 0.234. The majority of PIC values fell between 0.2 and 0.3, and 754,284 SNPs had a PIC greater than 0.25. Furthermore, the mean value of HE (0.351) was higher than that of HO (0.277), suggesting the presence of potential inbreeding or selection pressures within the population.

### 3.4. ROH Statistics and Inbreeding Coefficient

**Figure 3:**
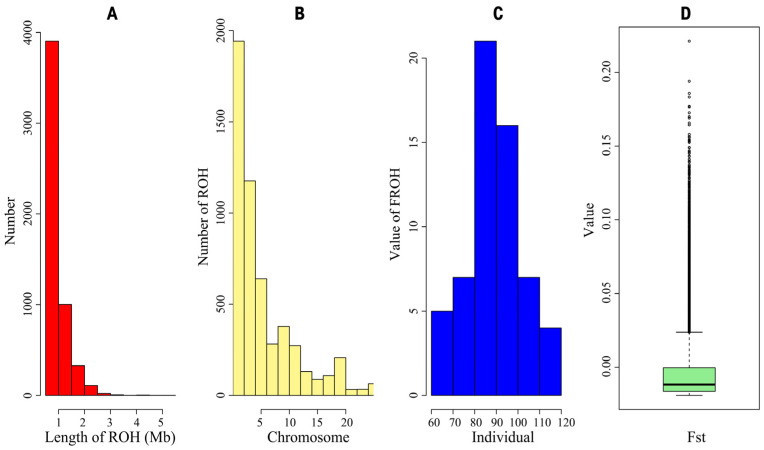
(**A**) Distribution of length of runs of homozygosity (ROH); (**B**) distribution of number of ROH across chromosomes; (**C**) distribution of value of FROH across individuals; (**D**) boxplot of Fixation index (FST) values within *Luhua chicken* population.

The distribution of ROHs reflects both the population’s genetic diversity and inbreeding levels. A substantial number of ROHs were identified, with the majority ranging in length from 0.5 to 1 Mb and only a few exceeding 3 Mb. The distribution of ROHs across chromosomes (Figure 3B) showed that Chromosomes 1 and 2 had the highest number of ROHs, approximately 1943, while Chromosomes 21 and 22 had the fewest. The inbreeding coefficient, calculated from runs of homozygosity (FROH), revealed varying levels of inbreeding across the population, with an overall FROH value of 0.012. Additionally, the FST values ranged from −0.019 to 0.221, with a mean of −0.012.

### 3.5. IBS Distance Matrix and G-Matrix Analysis

**Figure 4:**
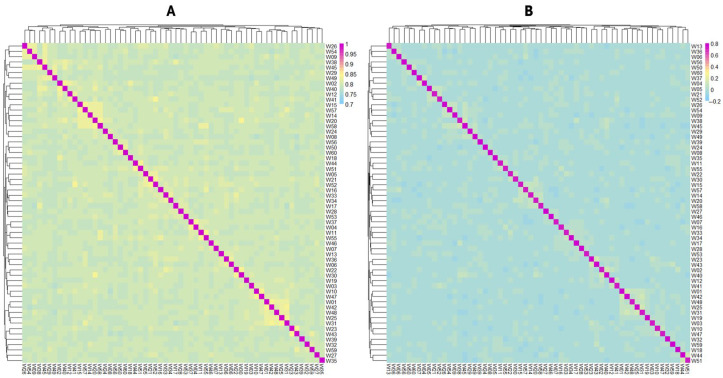
(**A**) Heat maps of identical-by-state distance matrix; (**B**) heat maps of genetic relationship matrix. Colors transitioning from blue to red indicate low-to-high genetic distance and genetic relationship.

The heat maps of the G-matrix and IBS distance matrix are shown in Figure 4. The IBS genetic distances of the population ranged from 0.771 to 0.845, with a mean of 0.782, indicating that most individuals were genetically distant from one another. The G-matrix revealed that most individuals in the population showed moderate kinship, while a few showed closer genetic relationships. Both the IBS distance matrix and G-matrix results indicated a potential risk of inbreeding.

### 3.6. Genetic Structure Analysis

**Figure 5:**
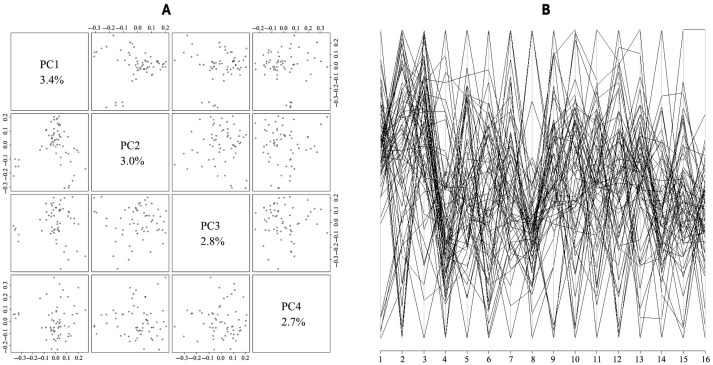
The population structure among 60 *Luhua chickens* demonstrated by principal component analysis (PCA). The population structure is demonstrated by the scatter plots, plotted on the axes of feature vectors PC1, PC2, PC3, and PC4 (**A**), and using a parallel coordinate plot for the top principal components (**B**).

To analyze the genetic structure of *Luhua chickens*, the population structure was analyzed using PCA (Figure 5). The first four PCs accounted for 3.4%, 3.0%, 2.8%, and 2.7% of the variation, respectively.

**Figure 6:**
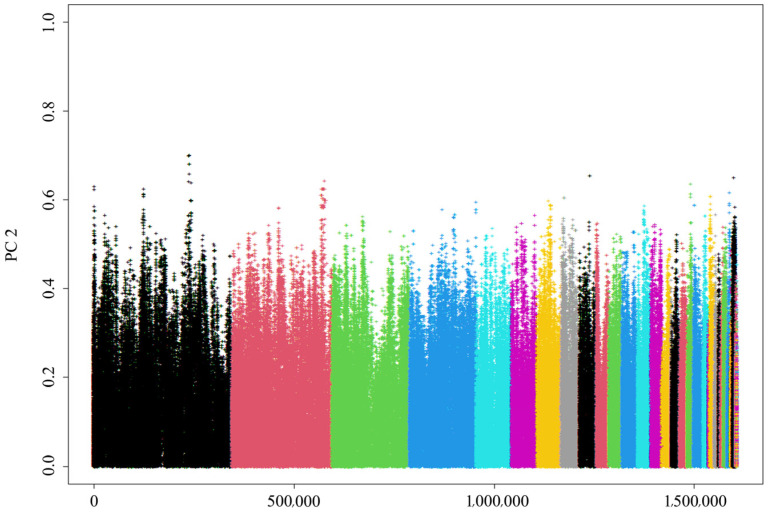
PCA of genetic variation across chromosomes that reflects the genetic structure and variation within the sampled population. Different colors represent distinct chromosomes.

The weak association between population variation, as explained by the principal components, and SNP genotypes (Figure 6) suggests that the distribution of SNPs was generally uniform. No obvious population stratification was found.

**Figure 7:**
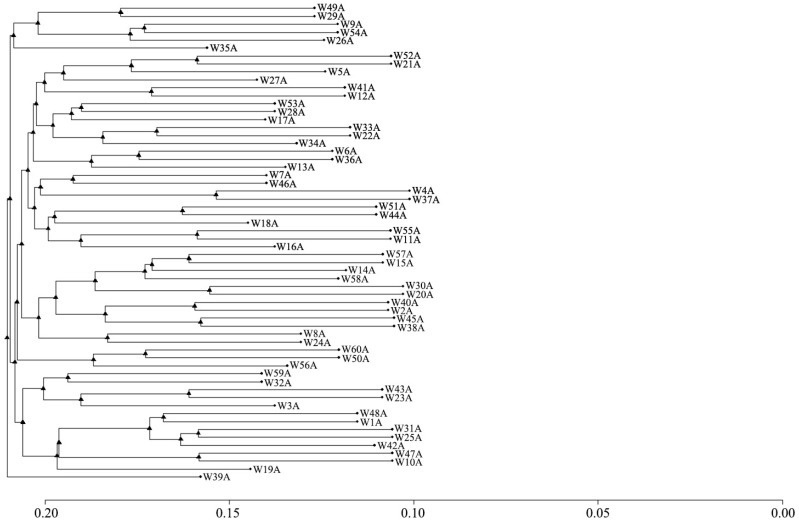
Dendrogram of relationships among 60 *Luhua chickens* revealed by genome-wide markers. The label for each branch corresponds to individual *Luhua chickens*. The *x*-axis represents the distance scale.

The clustering results are shown in Figure 7. The 60 *Luhua chickens* were preliminarily classified into nine grades. Among them, there were two individuals classified as grade nine, and there were nine individuals below grade eight. From this, it could be seen that the inbreeding phenomenon in the *Luhua chicken* population was not serious.

## 4. Discussion

The *Luhua chicken* is the only local chicken breed in China with characteristic Luhua feathers, and it is an important germplasm resource. It has been preserved due to the increasing emphasis on the conservation of genetic resources. At present, most local chicken conservation farms in China utilize closed breeding systems, which significantly affects the genetic diversity and structure. Therefore, elucidating the genetic diversity of local chicken conservation populations is crucial for the sustainable development of these populations. From an absolute numerical perspective, the phenotypic traits of *Luhua chickens* across three consecutive generations indicated that body weight at first egg increased, age at first egg was delayed each year, and the egg weight at first laying increased, which could be attributed to the delayed age at first egg [15]. Moreover, little variation in body weight at 300 d was seen over the three generations, and the total egg number per generation was similar, suggesting that the conservation status of *Luhua chicken* is stable.

In this study, SNP molecular markers proved to be essential for analyzing the genetic diversity of the *Luhua chicken* population, owing to their high coverage density, wide distribution, and genetic stability [16]. Through genome sequencing and quality control, 1,607,248 eligible SNPs were obtained. We applied stringent SNP filtering criteria (MAF < 0.05, call rate < 90%) to ensure data quality and remove potentially low-quality or unreliable genetic markers. While these criteria can help to improve the accuracy of the data, we also acknowledge that such filtering may result in the loss of rare alleles, which are crucial for assessing genetic diversity. Rare alleles often constitute an important part of population genetic diversity and play a key role in species adaptability and evolutionary potential [17]. SNP information was uniformly distributed across chromosomes. Specifically, Chromosome 1 (Chr1) displayed a high SNP density within the 153–156 Mb range. Chromosomes 2, 3, 4, and 5 also exhibited significant SNP densities in similar patterns, while chromosomes such as Chr5 and Chr8 showed partial deficiencies. This distribution indicates that certain chromosomal regions harbor greater genetic variability, which may be important for subsequent functional analyses [18].

Using the whole-genome resequencing data from *Luhua chickens*, the PIC, HE, and HO were analyzed. The results indicate that the HE (0.351) was higher than the HO (0.277), suggesting possible selection pressure or inbreeding within the population. This HE level, while indicating some diversity, is notably lower than that reported for broader collections of Chinese indigenous breeds or commercial Korean lines but is comparable to HE values found in some specific indigenous populations, such as certain Tibetan chickens (influenced by isolation and environment) [19] or certain Henan breeds (SNP-based HE 0.27–0.29). The reduced genetic diversity in *Luhua chickens* likely stems from a combination of factors, including historical protective breeding focused on specific traits, long-term isolation, limited gene flow, and the inherent effects of conservation management in closed populations, a phenomenon also observed in other conserved indigenous breeds [20]. Higher heterozygosity implies richer genetic diversity in the population. Compared with studies on other local chicken breeds, such as nine local chicken breeds in China (0.662) and the commercial Korean native chicken population (0.741) [21,22], the genetic diversity of *Luhua chickens* has decreased significantly, which may be due to a small population, causing the loss of some genes. The PIC values provide insight into the informativeness of the markers used, showing that most of the SNP loci had low polymorphisms, with PIC values concentrated between 0.2 and 0.3. Additionally, the concentration of MAFs between 0 and 0.2 pointed to the presence of low genetic variation within the population [23]. These findings could be linked to long-term breed isolation and limited gene flow, contributing to the population’s reduced genetic diversity [24]. In future studies, we will discuss the changes in population genetic statistics under ex situ conservation conditions.

The IBS genetic distances were used to assess relatedness among individuals. The IBS values of the *Luhua chicken* population ranged from 0.771 to 0.845, with a mean of 0.782, indicating that most individuals in the population have a relatively distant genetic relationship, with only a few individuals showing closer genetic relationships. By constructing a G-matrix for further analysis, we found that a small portion of individuals had a closer genetic relationship. The length and number of ROHs reflect information on the genomic structure and inbreeding levels of different populations. In this study, most ROHs were between 0.5 and 1 Mb in length, and only a few ROHs exceeded 3 Mb, suggesting that the population, in general, did not experience severe inbreeding [25]. However, the more concentrated distribution of ROHs on certain chromosomes, such as Chromosomes 1 and 2, implied that these regions might be under selective pressure or contain functional genes related to important traits. The high SNP density in these specific regions might reflect specific adaptive traits or economic traits in *Luhua chickens* and is a key direction for future research.

To further explore the population structure, PCA and cluster analysis were used, demonstrating that the *Luhua chicken* population was relatively homogeneous. This indicates that individuals within the population shared similar genetic backgrounds, reinforcing the conclusion of limited genetic diversity. While the initial principal component analysis (PCA) may explain only a small fraction (~12%) of the total genetic variation, this is not uncommon for populations like *Luhua chickens*. Such low variance is often attributable to crucial genetic factors like historical bottlenecks, limited gene flow, and founder effects that occurred during the establishment of conservation flocks, as documented in various domestic animal populations [26]. Studies have shown that bottleneck events and restricted gene flow can significantly reduce genetic diversity, which may explain the low variance captured by PCA [27,28]. Additionally, populations that have not undergone intensive selective breeding may retain traces of these genetic limitations, further influencing the ability of PCA to capture the underlying structure [29]. However, the stratification seen in certain family lines, such as W39A, highlighted a need for careful management to maintain genetic diversity and avoid excessive inbreeding within specific lines. Using balanced mating strategies is essential to maintain the genetic health of the population and prevent the loss of specific family lines [30]. This suggests that a rational mating program is needed to ensure the family structure remains balanced to avoid loss of families.

Overall, the genetic diversity of the *Luhua chicken* population is relatively low, and there is a potential risk of inbreeding compared with other local chicken breeds [31]. Therefore, to better conserve and utilize this genetic resource, it is essential to enhance the selection and mating processes, avoiding the mating of closely related individuals. Based on the results of the cluster analysis, the mating of families with close genetic distance should be minimized during the conservation process. This approach will be effective in reducing the inbreeding coefficient in the offspring of the conservation population, thereby promoting the population’s sustainable development.

## 5. Conclusions

This study was the first to explore the genetic diversity, ROH, and population structure of the *Luhua* breed of chickens. Our genetic diversity and population structure analysis revealed that it is a population with relatively low diversity and a potential risk of inbreeding. Genetic parameters such as the expected heterozygosity and polymorphism information content indicate limited genetic variation, primarily due to long-term isolation and conservation strategies. However, phenotypic traits such as body weight at first egg and egg weight showed no changes over generations, suggesting successful adaptation within the conservation program. To ensure the long-term viability of the *Luhua chicken* population, it is recommended that conservation efforts focus on optimizing breeding strategies to reduce inbreeding risks and promote genetic diversity. This research provides crucial insights for the effective management of genetic resources for indigenous chicken breeds.

## Figures and Tables

**Table 1:** Descriptive statistics for phenotypic data of *Luhua chickens*.

Traits	Generation	*n*	Mean	SD	95% Confidence Intervals	
					Upper Limit	Lower Limit
Body weight at first egg (g)	1	903	1452.99	173.97	1464.66	1441.32
	2	1070	1490.35	183.18	1501.37	1479.34
	3	1180	1512.57	167.85	1522.17	1502.96
Age at first egg (d)	1	903	161.97	9.10	162.58	161.36
	2	1070	166.71	15.17	167.62	165.80
	3	1180	174.86	14.34	175.68	174.05
First egg weight (g)	1	903	35.95	3.67	36.19	35.70
	2	1070	37.93	4.27	38.18	37.67
	3	1180	39.88	3.83	40.10	39.67
Body weight at 300 d (g)	1	903	1630.54	232.21	1645.98	1615.09
	2	1070	1647.38	232.65	1661.78	1632.97
	3	1180	1638.68	218.17	1651.37	1625.99
Total egg number	1	903	195.62	30.80	197.89	193.35
	2	1070	195.50	42.31	198.36	192.64
	3	1180	194.66	25.30	196.42	192.90

## Data Availability

The original contributions presented in this study are included in the article. Further inquiries can be directed to the corresponding author.
